# Association Between Physical Activity Levels in the Hospital Setting and Hospital-Acquired Functional Decline in Elderly Patients

**DOI:** 10.1001/jamanetworkopen.2019.20185

**Published:** 2020-01-31

**Authors:** Plamena Tasheva, Peter Vollenweider, Vanessa Kraege, Guillaume Roulet, Olivier Lamy, Pedro Marques-Vidal, Marie Méan

**Affiliations:** 1 Department of Medicine, Division of Internal Medicine, Lausanne University Hospital, Lausanne, Switzerland; 2 University of Lausanne, Lausanne, Switzerland

## Abstract

**Importance:**

The effects of in-hospital physical activity (PA) on outcomes among elderly patients has seldom been assessed.

**Objectives:**

To assess PA levels among elderly patients hospitalized for acute medical illness and to examine the association between PA levels and functional decline and other clinical outcomes at discharge.

**Design, Setting, and Participants:**

This monocentric cohort study was performed among patients 65 years or older who were admitted for acute medical illness to the internal medicine ward of Lausanne University Hospital, Lausanne, Switzerland, from February 1 through November 30, 2018. Data were analyzed from January 1 through December 2, 2019.

**Exposures:**

Daytime and 24-hour PA levels assessed via wrist accelerometers and measured in millig units (mG; 1 mG = 9.80665 × 10^−3^ m/s^2^).

**Mean Outcomes and Measures:**

Functional decline (defined as a ≥5-point decrease in the modified Barthel Index), risk of bedsores, length of stay (LOS), and inability to return home.

**Results:**

A total of 177 patients (106 [59.9%] men; median age, 83 [interquartile range, 74-87] years) were included. Lower mean (SD) PA levels were found in patients using walking aids before admission (daytime, 12 [5] vs 15 [7] mG; 24-hour, 10 [3] vs 11 [5] mG), those admitted for a reason associated with functional decline (daytime, 12 [6] vs 14 [7] mG; 24-hour, 10 [4] vs 11 [4] mG), or those prescribed physiotherapy (daytime, 12 [5] vs 15 [7] mG; 24-hour, 10 [4] vs 12 [5] mG). At discharge, functional decline was found in 63 patients (35.6%; 95% CI, 25.6%-43.1%), bedsore risk in 78 (44.1%; 95% CI, 36.6%-51.7%), and inability to return home in 82 (46.3%; 95% CI, 38.8%-54.0%). After multivariate analysis, no association was found between PA levels and functional decline (multivariable-adjusted mean [SE], 13 [1] vs 13 [1] mG for daytime levels [*P* = .69] and 10 [1] vs 11 [1] mG for 24-hour PA levels [*P* = .45]) or LOS (Spearman rank correlation, ρ = −0.06 for daytime PA levels [*P* = .93] and −0.01 for 24-hour PA levels [*P* = .52]). Patients at risk of bedsores had significantly lower PA levels than those not at risk (multivariable-adjusted mean [SE], 12 [1] vs 15 [1] mG for daytime PA levels [*P* = .008]; 10 [1] vs 12 [1] mG for 24-hour PA levels [*P* = .01]). Patients able to return home had significantly higher PA levels than those institutionalized (multivariable-adjusted mean [SE], 14 [1] vs 12 [1] mG for daytime PA levels [*P* = .04]; 11 [1] vs 10 [1] mG for 24-hour PA levels [*P* = .009]).

**Conclusions and Relevance:**

In this study, lower in-hospital daytime and 24-hour PA levels were associated with risk of bedsores and inability to return home on discharge. These findings are important given that one-third of elderly patients present with hospital-acquired functional decline.

## Key Points

**Question:**

What is the association between in-hospital physical activity and hospital-acquired functional decline in elderly patients?

**Findings:**

This cohort study included 177 patients 65 years or older, of whom 63 (35.6%) presented with functional decline; no association was found between physical activity levels and functional decline. Patients able to return home had significantly higher physical activity levels than those institutionalized (multivariable-adjusted mean, 14 vs 12 mG for daytime levels and 11 vs 10 mG for 24-hour levels).

**Meaning:**

In this study, many elderly hospitalized patients presented with functional decline on discharge, and higher in-hospital physical activity levels were inversely associated with institutionalization but were not associated with functional decline.

## Teaser

This cohort study assesses physical activity levels among elderly patients hospitalized for acute medical illness and examines the association between physical activity in the hospital setting and functional decline.

## Introduction

The presence of low physical activity (PA) levels among elderly hospitalized patients is an underrecognized epidemic.^1^ Indeed, during hospitalization for acute illness, 16% to 33% of elderly patients perform very little or no PA.^2,3^ Moreover, most elderly patients who are able to walk independently before admission spend most of their time (83%) lying in bed.^1^ Low PA levels decrease muscle strength and mass,^4^ and several studies have shown that inactivity during hospitalization is associated with a wide range of negative outcomes, such as hospital-acquired functional decline, increased risk of falls, longer length of hospitalization, and new institutionalization.^2,4,5,6^

Measurement of PA in the hospital setting relies mostly on qualitative nurse^3^ or physician^7^ perception and is poorly documented in hospital electronic records,^8^ thus limiting its clinical usefulness. Recently, the use of accelerometers allowed the collection of accurate, objective, and continuous PA data in hospitalized patients.^7,9,10,11,12,13,14,15,16,17^ Irrespective of the PA data collected (mean acceleration, steps count, gait velocity, and time of inactivity), all studies performed in the hospital setting confirmed that PA intensity and amount is low among elderly inpatients.^10,11,15,17^

Many of the previous studies were mostly descriptive and did not link objectively assessed PA levels with functional outcomes at hospital discharge. To our knowledge, only 1 study including 177 elderly patients assessed the association between step count and hospital-acquired functional decline.^18^ However, as in most studies, Agmon et al^18^ excluded elderly patients with cognitive impairment, a population at increased risk of posthospitalization adverse outcomes.^19^ Hence, the present study aimed to (1) assess PA levels among elderly patients hospitalized for acute medical illness and (2) examine the association between PA levels and functional decline and other clinical outcomes at discharge.

## Methods

### Setting

The NEXT-STEP Study was conducted from February 1 through November 30, 2018, in a 21-bed internal medicine ward of Lausanne University Hospital, Lausanne, in the canton of Vaud in the French-speaking part of Switzerland. Lausanne University Hospital has more than 1500 beds and admits more than 50 000 patients per year. The ethics committee of the canton of Vaud approved the study. The study was performed in agreement with the Declaration of Helsinki and its former amendments^20^ and in accordance with the applicable Swiss legislation. All participants or their legal representatives (in case of confusion or dementia) provided signed informed consent before entering the study. If a participant decided to withdraw from the study, data collected until the moment of withdrawal were used. This study followed the Strengthening the Reporting of Observational Studies in Epidemiology (STROBE) reporting guideline for cohort studies.

### Recruitment Procedure

Patients were recruited daily. Briefly, all patients 65 years or older admitted to the study ward directly or via the emergency unit were considered eligible. Participants were excluded if they (1) had a probable life expectancy of less than 30 days, based on clinical judgment (eg, progressive oncological disease or terminal heart failure); (2) had insufficient comprehension of the French language; (3) were unable to stand on their feet 1 week before hospitalization, as assessed by interview; or (4) were forced to bed rest by factors not directly related to the disease (eg, fracture). The selection procedure was applied within the first 3 days of hospitalization. If exclusion criteria were not met, patients were invited to participate, and the study procedure was explained. If the patient accepted, written informed consent was signed before the start of the study.

### PA Level Measurement

Physical activity level was assessed using a wrist accelerometer (GENEActiv Original; ActivInsights, Ltd), parametrized at 50 Hz. These accelerometers have been shown to provide a reliable and valid measurement of PA level in adults^21^ and hospitalized elderly patients.^9,16^ The devices were provided to the patients immediately after inclusion, and patients could choose on which wrist they preferred to wear it. Previous studies have shown that measuring site does not influence measurement.^22^ Patients were asked to wear the device continuously (day and night, including showering) until hospital discharge or transfer to another department (eg, intensive care or surgery unit).

Accelerometry data were extracted and analyzed using the GGIR package for R, version 9.1 (R Project for Statistical Computing).^23^ A valid daytime measurement was defined as at least 10 hours of daytime wear, and at least 24 hours of valid data were required for analysis (24-hour measurement).^24^ The GGIR algorithm allows the extraction of the mean daytime and 24-hour PA level, expressed as millig units (mG) (ie, variables Acc_day_milli-G_pla and ACC_nightandday_milli-G_pla of the GGIR output, respectively).

### Covariates

Covariates were extracted from the hospital electronic health record. These covariates included demographic data, comorbidities, use of benzodiazepines and antihypertensive drugs on admission, and prescription (yes or no) of physiotherapy. Comorbidities were used to compute the Charlson comorbidity index as described previously.^25^ Self-reported functional status, use of walking aids and history of falls during the year before admission, use of medical equipment (ie, urinary catheter or oxygen therapy), and isolation precautions at admission were collected by questionnaire.

Functional status was assessed at admission and discharge using the modified Barthel Index (BI) in a face-to-face interview. The BI has been reported as being the best scale to assess activities of daily living (ADL).^26^ The BI has a widespread use, and the modified version improves the internal consistency and provides better discrimination of functional ability.^26^ For patients with dementia or confusion, functional status before the hospitalization was assessed by interviewing their relatives or caregivers in face-to-face interviews or by telephone call. The patient’s functional capacity to perform 10 ADL tasks (ie, feeding, bathing, personal toileting, dressing and undressing, bowel and bladder control, getting on and off the toilet, moving from chair to bed and back, walking on level surfaces, and ascending and descending stairs) was rated as fully independent, uses minimal or moderate help, attempts task but unsafe, and unable to perform. Maximum BI was 100. A total BI of 0 to 20 suggests total dependence; 21 to 60, severe dependence; 61 to 90, moderate dependence; and 91 to 99, slight dependence. A score of 100 indicates that the patient is independent of assistance from others.

### Primary and Secondary Outcomes

Our main outcome was hospital-acquired functional decline, defined as a decrease or worsening of at least 5 points in the modified BI at discharge.^18,27^ This decrease is equivalent to full dependency in 1 ADL task among the 10 rated.

Our secondary outcomes were risk of bedsores on discharge, length of stay (LOS), number of falls during hospitalization, and inability to return home at hospital discharge (ie, need of rehabilitation or new institutionalization). Risk of bedsores was assessed using the Braden scale at inclusion and at discharge.^28^ The Braden scale rates patients in the following 6 subscales: Sensory Perception, Moisture, Activity, Mobility, Nutrition, and Friction and Shear. The maximum score is 23; a score 18 or less is indicative of risk of sore development. Occurrence and number of falls during the stay, LOS (in days), and inability to return home were extracted from the hospital electronic health record. Due to the skewed distribution of LOS, log-transformed values were used in the analysis.

### Sample Size Calculation

Data regarding hospital-acquired functional decline and prevalence of falls were obtained from the literature.^2,18,29,30,31,32^ Because patients were categorized into 2 groups of equal size (less than the median vs greater than or equal to the median), the minimum detectable effect sizes were calculated based on the following assumptions: power of 80%, α = 5%, equal sample sizes, proportions analyzed using bilateral χ^2^ test, and an attrition rate of 100%. The resulting sample size was 200, which allowed the detection of absolute differences in functional decline and falls of 19% and 18%, respectively. These values are lower than reported in the literature (37% and 23%, respectively).^18,30^

### Statistical Analysis

Data were analyzed from January 22 through December 2, 2019. Statistical analysis was conducted using Stata, version 15.1 (StataCorp LLC). Results were expressed as number (percentage) of patients for categorical variables and as mean (SD) for continuous variables. Between-group comparisons were performed using the χ^2^ test or the Fisher exact test for categorical variables and analysis of variance for continuous variables. The bivariate associations between PA levels and raw LOS were assessed using Spearman rank correlation. Multivariate analysis comparing PA levels according to categorical outcomes was performed using analysis of variance, and the results were expressed as multivariate-adjusted mean (SE). The bivariate and multivariate analysis assessing the association between PA levels and log-transformed LOS was performed using linear regression, and the results were expressed as slope (95% CI). A secondary analysis testing a U-shaped association was performed by introducing a quadratic term in the models. For multivariate analyses, 2 models were used: the first adjusted for sex and age and the second for sex, age, and BI at admission.

The main analysis included all patients with accelerometry data. A sensitivity analysis was conducted after excluding patients who had a nonwear time of greater than 20%. For patients with dementia or confusion, the functional status before hospitalization was obtained from relatives or caregivers, and a second sensitivity analysis without those patients was performed. Statistical significance was assessed for a 2-sided test with a *P* < .05.

## Results

### Sample Selection

Of the 377 initial patients approached, 274 were considered eligible for the study, and 177 (64.6%) were retained for analysis (106 men [59.9%] and 71 women [40.1%]; median age, 83 [interquartile range, 74-87] years). The selection procedure and reasons for exclusion are indicated in the eFigure in the Supplement. The characteristics of eligible patients who participated vs those who did not are summarized in eTable 1 in the Supplement. Compared with participants, nonparticipants were more frequently women (38 of 63 [60.3%] who did not consent), whereas no difference was found for other characteristics. Mean (SD) LOS of participants included in the study was 7.5 (5.2) days, with a median of 6 days and an interquartile range of 4 to 9 days.

### PA Levels According to Patient Characteristics

The daytime and 24-hour PA levels according to the characteristics of patients are summarized in Table 1. Lower mean (SD) PA levels were found among patients using walking aids before admission (daytime, 12 [5] vs 15 [7] mG; 24-hour, 10 [3] vs 11 [5] mG), admitted for a reason associated with functional decline (daytime, 12 [6] vs 14 [7] mG; 24-hour, 10 [4] vs 11 [4] mG), or prescribed physiotherapy (daytime, 12 [5] vs 15 [7] mG; 24-hour, 10 [4] vs 12 [5] mG). Lower mean (SD) daytime PA levels were found among patients with cognitive impairment (11 [4] vs 14 [7] mG) or at risk of bedsores on inclusion (Braden score <18) (12 [6] vs 14 [6] mG), whereas no differences were found for 24-hour PA levels.

**Table 1.** Mean Daytime and 24-Hour Acceleration According to Patient Characteristics^a^

Characteristic	No. of Patients	Mean (SD) Acceleration, mG	
		Daytime	24 h
**Physical Characteristics**			
Sex			
Men	106	13 (6)	11 (4)
Women	71	13 (6)	10 (4)
*P* value	NA	.99	.77
Depressive disorders			
No	151	13 (6)	11 (4)
Yes	26	12 (5)	10 (3)
*P* value	NA	.52	.44
Urinary and/or fecal incontinence			
No	122	14 (7)	11 (5)
Yes	55	12 (5)	10 (4)
*P* value	NA	.17	.26
Hearing loss and/or vision issues			
No	146	13 (6)	10 (4)
Yes	31	14 (6)	11 (4)
*P* value	NA	.62	.61
**Anamnesis**			
Walking aids 2 weeks before admission			
No	87	15 (7)	11 (5)
Yes	90	12 (5)	10 (3)
*P* value	NA	.002	.01
History of falls before admission			
No	54	12 (6)	10 (4)
Yes	123	14 (6)	11 (4)
*P* value	NA	.18	.46
Reason for admission associated with functional decline^b^			
No	86	14 (7)	11 (4)
Yes	91	12 (6)	10 (4)
*P* value	NA	.007	.02
**Status at Inclusion**			
Cognitive impairment and/or confusion			
No	125	14 (7)	11 (5)
Yes	52	11 (4)	10 (3)
*P* value	NA	.02	.17
Sedative drugs used^c^			
No	149	13 (6)	10 (4)
Yes	27	14 (5)	11 (3)
*P* value	NA	.27	.43
Braden score^c^^,^^d^			
≤18	90	14 (6)	11 (4)
>18	81	12 (6)	10 (4)
*P* value	NA	.01	.06
Medical equipment used^e^			
No	141	14 (6)	11 (4)
Yes	36	12 (6)	10 (4)
*P* value	NA	.10	.15
Isolation precautions			
No	171	13 (6)	10 (4)
Yes	6	15 (5)	12 (3)
*P* value	NA	.58	.43
Prescription of physiotherapy			
No	64	15 (7)	12 (5)
Yes	113	12 (5)	10 (4)
*P* value	NA	.002	.02

Abbreviation: NA, not applicable.

^a^ Data are from the NEXT-STEP Study, Lausanne, Switzerland, February 1 through November 30, 2018. Between-group comparisons were conducted using analysis of variance.

^b^ Includes gait problems or fall, general state alteration, musculoskeletal pain, and neurological deficit.

^c^ Data were missing for some patients.

^d^ A score of 18 or less indicates risk of bed sore development.

^e^ Includes urinary catheter or oxygen therapy.

### Association Between PA Levels and Outcomes

At discharge, a worsening of the BI was found in 63 patients (35.6%; 95% CI, 25.6%-43.1%) (Figure); risk of bedsores (ie, a Braden score <18) was found in 78 patients (44.1%; 95% CI, 36.6%-51.7%); inability to return home occurred in 82 patients (46.3%; 95% CI, 38.8%-54.0%); and in-hospital falls occurred in 3 patients (1.7%; 95% CI, 0.4%-4.9%). The bivariate and multivariate associations between PA levels and categorical outcomes are summarized in Table 2. On bivariate analysis, lower mean (SE) PA levels were found in patients at risk of bedsores upon discharge (daytime, 11 [5] vs 15 [7] mG; 24-hour, 9 [4] vs 12 [5] mG) or unable to return home (daytime, 12 [5] vs 14 [7] mG; 24-hour, 10 [4] vs 11 [5] mG), and this difference persisted after multivariate adjustment, whereas no association was found between PA levels and functional decline (multivariable-adjusted mean [SE], 13 [1] vs 13 [1] mG for daytime levels [*P* = .69] and 10 [1] vs 11 [1] mG for 24-hour PA levels [*P* = .45]). Patients at risk of bedsores had significantly lower PA levels than those not at risk (multivariable-adjusted mean [SE], 12 [1] vs 15 [1] mG for daytime PA levels [*P* = .008]; 10 [1] vs 12 [1] mG for 24-hour PA levels [*P* = .01]). Patients able to return home had significantly higher PA levels than those institutionalized (multivariable-adjusted mean [SE], 14 [1] vs 12 [1] mG for daytime PA levels [*P* = .04]; 11 [1] vs 10 [1] mG for 24-hour PA levels [*P* = .01]) (Table 2).

**Figure.**
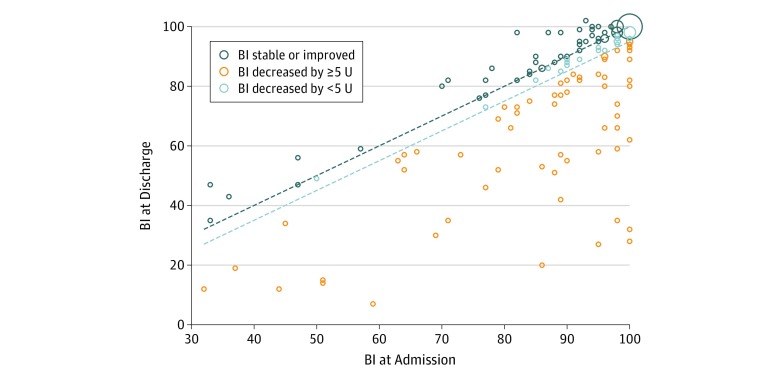
Association Between Barthel Index (BI) at Admission and Discharge The area of the markers is proportional to the number of patients. Markers above the dark blue dashed line correspond to patients whose BI remained stable or improved. The dark blue dashed line corresponds to unity (BI at discharge equals BI at admission); the light blue dashed line corresponds to a decrease in 5 U in the BI at discharge relative to the BI at admission. Markers below the light blue dashed line correspond to patients whose BI decreased by 5 U or more. Light blue markers between the dashed lines correspond to patients whose BI decreased by less than 5 U between admission and discharge. The BI ranges from 0 (total dependence) to 100 (independence).

**Table 2.** Mean Daytime and 24-Hour Acceleration According to Outcomes^a^

Outcome	No. of Patients	Mean Acceleration, mG	
		Daytime	24 h
**Bivariate Analysis**			
Worsening of BI			
No	114	14 (6)	11 (4)
Yes	63	12 (6)	10 (4)
*P* value	NA	.23	.18
Risk of sores at discharge			
No	75	15 (7)	12 (5)
Yes	78	11 (5)	9 (4)
*P* value	NA	<.001	<.001
Falls			
No	4	13 (6)	11 (4)
In-hospital	167	12 (6)	10 (4)
*P* value	NA	.77	.80
Discharge disposition			
Return home	93	14 (7)	11 (5)
Inability to return home	82	12 (5)	10 (4)
*P* value	NA	.005	.002
**First Multivariate Analysis**^**b**^			
Worsening of BI			
No	114	14 (1)	11 (1)
Yes	63	13 (1)	10 (1)
*P* value	NA	.31	.21
Risk of sores at discharge			
No	75	15 (1)	12 (1)
Yes	78	11 (1)	9 (1)
*P* value	NA	<.001	<.001
Falls			
No	4	NC	NC
In-hospital	167	NC	NC
*P* value	NA	NA	NA
Discharge disposition			
Return home	93	14 (1)	12 (1)
Inability to return home	82	12 (1)	10 (1)
*P* value	NA	.01	.003
**Second Multivariate Analysis**^**c**^			
Worsening of BI			
No	114	13 (1)	11 (1)
Yes	63	13 (1)	10 (1)
*P* value	NA	.69	.45
Risk of sores at discharge			
No	75	15 (1)	12 (1)
Yes	78	12 (1)	10 (1)
*P* value	NA	.008	.01
Falls			
No	4	NC	NC
In-hospital	167	NC	NC
*P* value	NA	NA	NA
Discharge disposition			
Return home	93	14 (1)	11 (1)
Inability to return home	82	12 (1)	10 (1)
*P* value	NA	.04	.009

Abbreviations: BI, Barthel Index; NA, not applicable; NC, not computable.

^a^ Data are from NEXT-STEP Study, Lausanne, Switzerland, February 1 through November 30, 2018. For bivariate analyses, results are expressed as mean (SD); for multivariate analyses, results are expressed as adjusted mean (standard error); analyses were performed using analysis of variance.

^b^ Adjusted for sex and age.

^c^ Adjusted for sex, age, and BI at admission.

No associations were found between PA levels and raw LOS (Spearman rank correlation, ρ = −0.01 for daytime PA levels [*P* = .93] and −0.05 for 24-hour PA levels [*P* = .52]). The bivariate and multivariate associations between PA levels and log-transformed LOS are summarized in Table 3. No association was found between daytime or 24-hour PA levels and LOS, and no significant quadratic term was found. Similar findings were obtained when analyses were restricted to patients with less than 20% of accelerometer nonwear time (eTables 2 and 3 in the Supplement) or after excluding participants with dementia or confusion (eTables 4 and 5 in the Supplement).

**Table 3.** Association Between Mean Daytime and 24-Hour Acceleration and LOS^a^

LOS, Log-Transformed	Slope (95% CI)	
	Daytime	24 h
Bivariate	0.001 (−0.015 to 0.016)	−0.005 (−0.028 to 0.017)
Multivariate^b^	0.000 (−0.016 to 0.015)	−0.006 (−0.029 to 0.017)
Multivariate^c^	−0.001 (−0.017 to 0.016)	−0.007 (−0.030 to 0.017)

Abbreviation: LOS, length of stay.

^a^ Data are from NEXT-STEP Study, Lausanne, Switzerland, February 1 through November 30, 2018. Analyses were performed using linear regression.

^b^ Adjusted for sex and age.

^c^ Adjusted for sex, age, and Barthel Index at admission.

## Discussion

The purpose of this study was to assess PA levels of elderly patients in the hospital setting and to examine the association between PA levels and clinical outcomes on discharge. Our results show that PA levels do not appear to be associated with hospital-acquired functional decline. Conversely, patients not at risk of bedsores or those able to return home had higher daytime and 24-hour PA levels than patients at risk of bedsores or patients needing institutionalization.

### PA Levels According to Patient Characteristics

Mean PA levels for patients were low. This finding is consistent with existing literature^1,2,11,14,16^ and suggests that elderly patients do not perform PA in adequate amounts during their hospital stay, which leads to adverse clinical outcomes.^2,4,5,6^

Patients using walking aids before admission, admitted for a reason associated with functional decline, or prescribed physiotherapy had lower daytime and 24-hour PA levels than patients devoid of the conditions. The presence of a walking aid might impair the mobility of the patients, thus precluding them from moving adequately during hospitalization. Similarly, the presence of a functionally impairing condition might also aggravate the mobility of patients. More importantly, patients being prescribed physiotherapy had lower PA levels than those not prescribed physiotherapy. Physiotherapy has been shown to reduce functional decline among elderly hospitalized patients^33^; still, our results suggest that the amount of physiotherapy prescribed in this setting fails to increase PA to levels compatible with a beneficial outcome.

### Association Between PA Levels and Outcomes

No association was found between PA levels and hospital-acquired functional decline. Our results do not replicate the findings of 2 Israeli studies^18,19^ that assessed PA in elderly patients admitted to an internal medical unit using a self-reported questionnaire or accelerometry. Possible explanations include a higher mean BI at admission in our study (88 [SD, 16] compared with 78-85) and different methods of measuring PA (mean acceleration in millig units vs total number of steps per day or self-reported mobility levels).

Only 3 patients fell during the study period; this small number precluded any analysis between PA and falls. This number is not a hindrance per se, because a large number of falls indicates inadequate patient management and protection. Hence, this outcome might be of lesser interest in a context of good hospital practice, where active prevention of falls is standard care.

No association was found between PA levels and LOS. This finding does not replicate the previous literature; an Irish study conducted among 154 patients (mean [SD] age, 77.5 [7.4] years)^34^ found that a 50% increase in mean daily step count was associated with a 6% decrease in LOS. Another study^35^ reported that elderly patients who increased their walking to 600 steps per day (ie, approximately 12 minutes of slow walking) were discharged approximately 2 days earlier than patients who did not reach 600 steps per day. Because our study did not collect data allowing the calculation of the number of steps, comparisons with other studies are difficult. Still, our results suggest that spontaneous PA is not associated with LOS among elderly hospitalized patients, probably due to its very low intensity level.

Patients who were not at risk of bedsores on discharge had higher daytime and 24-hour PA levels than patients at risk. This finding is in agreement with that of a previous retrospective Portuguese study,^36^ in which low patient mobilization was associated with risk of sores independently of the total Braden score. Conversely, another study^37^ found no association between PA assessed by questionnaire and bedsores. A possible explanation is that PA was assessed by questionnaire instead of being objectively evaluated. Future studies should assess PA levels using objective rather than self-reported data.

Patients performing lower daytime and 24-hour levels of PA tended to be institutionalized more frequently. These findings agree with those of a previous study^38^ reporting that lower PA levels, assessed using a validated measure of patient mobility (Activity Measure for Post-Acute Care Inpatients Mobility Short Form), were associated with discharge placement. Indeed, recognition of low PA levels at admission or declining PA levels during hospital stay could prompt prescription of physiotherapy to address patients’ mobility issues.

### Recommendations for Future Studies

Our results appear to strengthen the available evidence that low PA levels are associated with unfavorable outcomes among hospitalized elderly patients. Future studies should try to estimate the minimum amount of PA needed to prevent increased in-hospital morbidity or LOS in elderly hospitalized patients. Assessment of PA levels using accelerometry requires specific equipment and software as well as adequate information technology knowledge to extract, process, and interpret the results. Hence, using accelerometers for routine assessment of PA levels among hospitalized patients is currently not recommended, and simpler methods, such as pedometers, should be preferred.^16^ Hence, further studies are needed to identify the motion sensor and the detection algorithm most suitable to assess PA levels in elderly hospitalized patients.

### Strengths and Limitations

The strengths of this study are its large sample size, its broad inclusion criteria (ie, including confused patients or use of walking aids before admission), the use of a validated accelerometer to assess PA, and the large number of activity hours recorded (3700 overall). This study also has several limitations. First, it was conducted in a single ward of a university hospital, and generalizability might be limited. Still, the characteristics of the patients correspond to those of most patients in most internal and general medicine wards. Second, there is a certain degree of interdependence between ADL and PA levels, which raises the issue of how to interpret the results. Activities of daily living are partly a measure of PA; thus, patients who are less able to engage in PA will also be less physically active in the hospital. Third, risk of bedsores was used instead of actual bedsores, because occurrence of bedsores in our department is very rare due to standard preventive measures. Had the number of bedsores been used, the group of interest would have been small, thus reducing statistical power.

## Conclusions

One-third of elderly patients present with hospital-acquired functional decline. In this study, lower in-hospital daytime and 24-hour PA levels were associated with risk of bedsores and inability to return home on discharge. No association was found between PA levels and hospital-acquired functional decline or LOS.

## Notes

Supplement. **eFigure.** Selection Procedure **eTable 1.** Characteristics of Patients Who Accepted and Did Not Accept Participation in the NEXT-STEP Study, Lausanne, Switzerland, 2018 **eTable 2.** Mean Acceleration During Daytime and the Whole Day According to Outcomes, Participants With ≥80% Accelerometry Wear Time **eTable 3.** Association Between Mean Acceleration During Daytime and the Whole Day and Length of Hospital Stay, Participants With ≥80% Accelerometry Wear Time **eTable 4.** Mean Acceleration During Daytime and the Whole Day According to Outcomes, Excluding Participants With Dementia or Confusion **eTable 5.** Association Between Mean Acceleration During Daytime and the Whole Day and Length of Hospital Stay, Excluding Participants With Dementia or Confusion
