# Supplementary material for: Association Between Physical Activity Levels in the Hospital Setting and Hospital-Acquired Functional Decline in Elderly Patients
Source: JAMA Netw Open. 2020 Jan 31;3(1):e1920185. doi: 10.1001/jamanetworkopen.2019.20185 (PMC7042865; doi:10.1001/jamanetworkopen.2019.20185)
Supplement: Supplement. — eFigure. Selection Procedure eTable 1. Characteristics of Patients Who Accepted and Did Not Accept Participation in the NEXT-STEP Study, Lausanne, Switzerland, 2018 eTable 2. Mean Acceleration During Daytime and the Whole Day According to Outcomes, Participants With ≥80% Accelerometry Wear Time eTable 3. Association Between Mean Acceleration During Daytime and the Whole Day and Length of Hospital Stay, Participants With ≥80% Accelerometry Wear Time eTable 4. Mean Acceleration During Daytime and the Whole Day According to Outcomes, Excluding Participants With Dementia or Confusion eTable 5. Association Between Mean Acceleration During Daytime and the Whole Day and Length of Hospital Stay, Excluding Participants With Dementia or Confusion [file jamanetwopen-3-e1920185-s001.pdf]

Supplementary Online Content

Tasheva P, Vollenweider P, Kraege V, et al. Association between physical activity levels in the hospital setting and hospital-acquired functional decline in elderly patients. *JAMA Netw Open.* 2020;3(1):e1920185. doi:10.1001/jamanetworkopen.2019.20185

eFigure. Selection Procedure

eTable 1. Characteristics of Patients Who Accepted and Did Not Accept Participation in the NEXT-STEP Study, Lausanne, Switzerland, 2018

eTable 2. Mean Acceleration During Daytime and the Whole Day According to Outcomes, Participants With ≥80% Accelerometry Wear Time

eTable 3. Association Between Mean Acceleration During Daytime and the Whole Day and Length of Hospital Stay, Participants With ≥80% Accelerometry Wear Time

eTable 4. Mean Acceleration During Daytime and the Whole Day According to Outcomes, Excluding Participants With Dementia or Confusion

eTable 5. Association Between Mean Acceleration During Daytime and the Whole Day and Length of Hospital Stay, Excluding Participants With Dementia or Confusion

This supplementary material has been provided by the authors to give readers additional information about their work.

© 2020 Tasheva P et al. *JAMA Network Open.*

eFigure. Selection Procedure

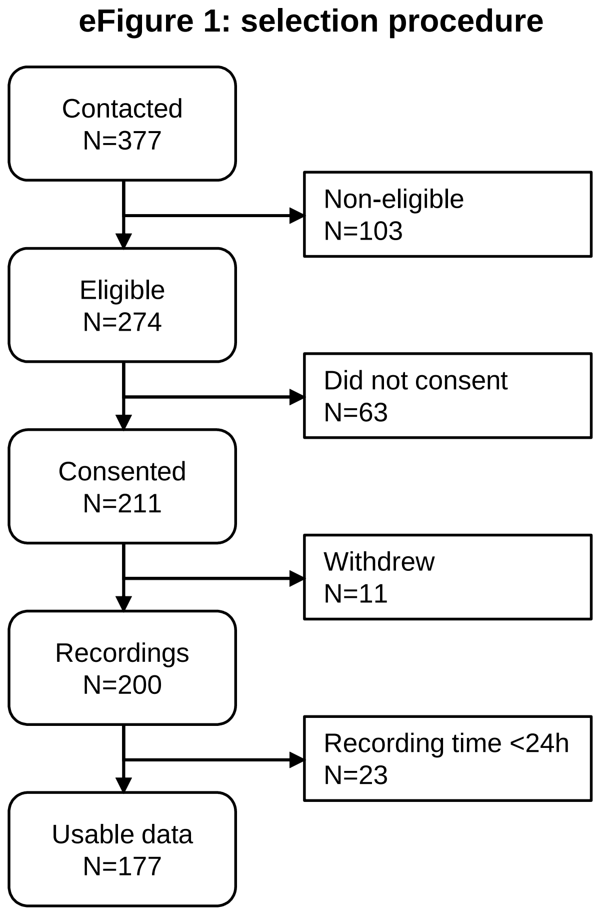

© 2020 Tasheva P et al. *JAMA Network Open.*

eTable 1. Characteristics of Patients Who Accepted and Did Not Accept Participation in the NEXT-STEP Study, Lausanne, Switzerland, 2018

	Yes	No	P-value
N	211	63	
Women (%)	89 (42.2)	38 (60.3)	0.011
Swiss nationality (%)	200 (94.8)	58 (92.1)	0.419
Clinical data upon admission (%)			
Gait problems/fall	54 (25.6)	14 (22.2)	0.587
General state alteration	71 (33.7)	29 (46.0)	0.073
Dyspnea	59 (28.0)	19 (30.2)	0.735
Musculoskeletal pain	9 (4.3)	1 (1.6)	0.463 §
Fever	26 (12.3)	5 (7.9)	0.335
Diarrhea / vomiting	20 (9.5)	6 (9.5)	0.991

Results are expressed as number of patients (column percentage). Statistical analysis by chi-square or Fisher's exact test (§).

© 2020 Tasheva P et al. *JAMA Network Open.*

eTable 2. Mean Acceleration During Daytime and the Whole Day According to Outcomes, Participants With ≥80% Accelerometry Wear Time

		N	Daytime	24-hour
Bivariate	No worsening of Barthel index	102	13.2 ± 5.8	10.5 ± 3.8
	Worsening of Barthel index	61	12.4 ± 6.3	10.0 ± 4.0
	P-value		0.406	0.384
	No risk of sores at discharge	67	15.0 ± 6.7	11.6 ± 4.3
	Risk of sores at discharge	77	11.3 ± 5.2	9.3 ± 3.5
	P-value		<0.001	<0.001
	No falls	3	12.7 ± 7.7	10.3 ± 4.7
	In-hospital falls	155	13.1 ± 6.0	10.4 ± 3.9
	P-value		0.910	0.956
	Return home	82	14.0 ± 6.3	11.1 ± 4.0
	Inability to return home	79	11.8 ± 5.5	9.5 ± 3.6
	P-value		0.021	0.012
Multivariable [a]	No worsening of Barthel index	102	13.2 ± 0.6	10.5 ± 0.4
	Worsening of Barthel index	61	12.5 ± 0.8	10.0 ± 0.5
	P-value		0.463	0.387
	No risk of sores at discharge	67	15.0 ± 0.7	11.6 ± 0.5
	Risk of sores at discharge	77	11.2 ± 0.7	9.3 ± 0.5
	P-value		<0.001	<0.001
	No falls	3	NC	NC
	In-hospital falls	155	NC	NC
	P-value		-	-
	Return home	82	14.0 ± 0.7	11.2 ± 0.4
	Inability to return home	79	11.8 ± 0.7	9.5 ± 0.4
	P-value		0.027	0.009
Multivariable [b]	No worsening of Barthel index	102	13.0 ± 0.6	10.4 ± 0.4
	Worsening of Barthel index	61	12.9 ± 0.8	10.2 ± 0.5
	P-value		0.915	0.723
	No risk of sores at discharge	67	14.5 ± 0.8	11.4 ± 0.5
	Risk of sores at discharge	77	11.7 ± 0.7	9.5 ± 0.5
	P-value		0.011	0.012
	No falls	3	NC	NC
	In-hospital falls	155	NC	NC
	P-value		-	-
	Return home	82	13.8 ± 0.7	11.0 ± 0.4
	Inability to return home	79	12.1 ± 0.7	9.6 ± 0.4
	P-value		0.072	0.024

[a], adjusting for gender and age; [b], adjusting for gender, age and Barthel index at admission. NC, not computable. For bivariate analyses, results are expressed as average ± standard deviation; for multivariable analyses, results are expressed as adjusted average ± standard error; analyses were performed using analysis of variance.

NEXT-STEP study, Lausanne, Switzerland, 2018.

© 2020 Tasheva P et al. *JAMA Network Open.*

eTable 3. Association Between Mean Acceleration During Daytime and the Whole Day and Length of Hospital Stay, Participants With ≥80% Accelerometry Wear Time

	**Daytime**	**24-hour**
LOS, log-transformed		
Bivariate	0.004 (-0.013 ; 0.020)	0.001 (-0.025 ; 0.027)
Multivariable [a]	0.003 (-0.014 ; 0.020)	0.001 (-0.025 ; 0.027)
Multivariable [b]	0.004 (-0.014 ; 0.021)	0.002 (-0.024 ; 0.029)

[a], adjusting for gender and age; [b], adjusting for gender, age and Barthel index at admission. LOS, length of stay. Results are expressed as slope and (95% confidence interval); analyses were performed using linear regression.

NEXT-STEP study, Lausanne, Switzerland, 2018.

© 2020 Tasheva P et al. *JAMA Network Open.*

eTable 4. Mean Acceleration During Daytime and the Whole Day According to Outcomes, Excluding Participants With Dementia or Confusion

		N	Daytime	24-hour
Bivariate	No worsening of Barthel index	88	14.1 ± 6.7	11.1 ± 4.7
	Worsening of Barthel index	37	13.2 ± 7.1	10.1 ± 4.4
	P-value		0.499	0.292
	No risk of sores at discharge	57	16.0 ± 7.7	12.1 ± 5.4
	Risk of sores at discharge	48	11.6 ± 5.8	9.4 ± 3.8
	P-value		0.002	0.004
	No falls	2	14.1 ± 10.4	10.7 ± 6.5
	In-hospital falls	119	14.0 ± 6.8	10.9 ± 4.7
	P-value		0.987	0.941
	Return home	70	15.0 ± 7.3	11.8 ± 5.0
	Inability to return home	54	12.3 ± 5.9	9.6 ± 3.8
	P-value		0.026	0.011
Multivariable [a]	No worsening of Barthel index	88	14.1 ± 0.7	11.1 ± 0.5
	Worsening of Barthel index	37	13.2 ± 1.1	10.1 ± 0.8
	P-value		0.549	0.319
	No risk of sores at discharge	57	16.0 ± 0.9	12.2 ± 0.6
	Risk of sores at discharge	48	11.5 ± 1.0	9.3 ± 0.7
	P-value		0.002	0.004
	No falls	2	NC	NC
	In-hospital falls	119	NC	NC
	P-value		-	-
	Return home	70	15.1 ± 0.8	11.8 ± 0.6
	Inability to return home	54	12.2 ± 0.9	9.5 ± 0.6
	P-value		0.027	0.010
Multivariable [b]	No worsening of Barthel index	88	13.8 ± 0.7	10.9 ± 0.5
	Worsening of Barthel index	37	13.8 ± 1.1	10.5 ± 0.8
	P-value		0.955	0.630
	No risk of sores at discharge	57	15.6 ± 1.0	11.9 ± 0.7
	Risk of sores at discharge	48	12.1 ± 1.1	9.7 ± 0.7
	P-value		0.024	0.042
	No falls	2	NC	NC
	In-hospital falls	119	NC	NC
	P-value		-	-
	Return home	70	14.8 ± 0.8	11.6 ± 0.6
	Inability to return home	54	12.7 ± 1.0	9.8 ± 0.6
	P-value		0.117	0.047

[a], adjusting for gender and age; [b], adjusting for gender, age and Barthel index at admission. NC, not computable. For bivariate analyses, results are expressed as average ± standard deviation; for multivariable analyses, results are expressed as adjusted average ± standard error; analyses were performed using analysis of variance.

NEXT-STEP study, Lausanne, Switzerland, 2018.

© 2020 Tasheva P et al. *JAMA Network Open.*

eTable 5. Association Between Mean Acceleration During Daytime and the Whole Day and Length of Hospital Stay, Excluding Participants With Dementia or Confusion

	Daytime	24-hour
LOS, log-transformed		
Bivariate	-0.003 (-0.020 ; 0.014)	-0.011 (-0.035 ; 0.014)
Multivariable [a]	-0.004 (-0.021 ; 0.014)	-0.011 (-0.036 ; 0.014)
Multivariable [b]	0.000 (-0.018 ; 0.017)	-0.007 (-0.032 ; 0.019)

[a], adjusting for gender and age; [b], adjusting for gender, age and Barthel index at admission. LOS, length of stay. Results are expressed as slope and (95% confidence interval); analyses were performed using linear regression.

NEXT-STEP study, Lausanne, Switzerland, 2018.

© 2020 Tasheva P et al. *JAMA Network Open.*
